# A 6–18 GHz High-Efficiency GaN Power Amplifier Using Transistor Stacking and Reactive Matching

**DOI:** 10.3390/mi17030338

**Published:** 2026-03-10

**Authors:** Cetian Wang, Xuejie Liao, Moquan Gong, Fei Xiao, He Guan, Fan Zhang, Deyun Zhou

**Affiliations:** 1 School of Microelectronics, Northwestern Polytechnical University, Xi’an 710129, China; wangct@ganide.com (C.W.); he.guan@nwpu.edu.cn (H.G.); dyzhou@nwpu.edu.cn (D.Z.); 2 Chengdu Ganide Technology Company, Ltd., Chengdu 610220, China; liaoxuejie@foxmail.com; 3 School of Electronic Science and Engineering, University of Electronic Science and Technology of China, Chengdu 610054, China; gongmq@std.uestc.edu.cn (M.G.); fxiao@uestc.edu.cn (F.X.)

**Keywords:** GaN, monolithic microwave integrated circuit (MMIC), power amplifier (PA), reactive matching, transistor stacking technology

## Abstract

This article presents the design and implementation of a 6–18 GHz GaN monolithic microwave integrated circuit (MMIC) power amplifier (PA). A two-stage cascaded reactive matching network structure based on transistor stacking technology is employed to achieve circuit gain, and a multi-cell combination is used in the final stage to simultaneously achieve high power and high efficiency. For demonstration, a prototype of the proposed PA with an area of 4.5 × 3.4 mm^2^ is fabricated in a 0.1 µm GaN-on-Si high-electron-mobility transistor (HEMT) process. The measured results of the GaN PA show a small signal gain of 25–29 dB, an output power of 40.8–42.5 dBm, and a power-added efficiency (PAE) of 27–38% in the operating frequency range of 6–18 GHz.

## 1. Introduction

With the rapid development of the millimeter-wave spectrum in various fields such as 5G communications, satellite communications, autonomous driving radar, and military electronic warfare, there is a growing demand for power amplifiers (PAs) that offer a broad bandwidth, high power, and high efficiency [1,2,3,4,5]. To meet this demand, III–V compound semiconductor monolithic microwave integrated circuits (MMICs) have become one of the key technologies for such PAs. Among these technologies, GaAs and InP perform well in high-frequency applications, but they have limitations in power density and thermal stability [6,7,8]. In contrast, GaN features a high power density, high breakdown voltage, and wideband capabilities, making it particularly suitable for the design of high-power, broadband power amplifiers [9]. Moreover, the GaN-on-Si (GaN/Si) process offers significant cost advantages over the GaN-on-SiC (GaN/SiC) process, making it a promising choice for achieving both a high performance and low cost [10,11]. In recent years, various PA architectures using GaN technology have been reported to cover the 6–18 GHz band, such as distributed [4,9,12], reactive matching [13], balanced [14,15,16], and push–pull configurations [17,18]. However, the main drawback of these traditional MMIC architectures is their relatively low power gain and efficiency.

Reactive matching networks enable low-loss broadband impedance transformation and are, therefore, well suited for wideband high-efficiency GaN PAs. Although stacked transistor architectures can enhance voltage handling and output power, their benefits in high-supply-voltage GaN technologies are not always straightforward due to parasitic effects and design complexity, motivating a careful co-design of stacking and broadband matching.

To address these challenges, this article presents a GaN stacked reactive matching power amplifier (SRMPA) operating over the 6–18 GHz frequency range. A reactive matching network is employed to enable low-loss and broadband impedance transformation. To enhance gain performance, a 4 × 3 stacked FET unit is used in the driver stage, while an 8 × 3 stacked FET unit is implemented in the power stage to maintain high output power and efficiency across the wide bandwidth. Fabricated using a GaN-on-Si HEMT process, the chip occupies a compact area of 4.5 × 3.4 mm^2^ and requires no additional off-chip matching components. The proposed PA delivers an average saturated output power exceeding 40.8 dBm and achieves a power-added efficiency (PAE) of 27–38% across the entire operating band.

The paper is organized as follows: Section 2 presents the design and analysis of the proposed power amplifier. Section 3 gives the experimental results and comparison. The conclusion is drawn in Section 4.

## 2. Broadband Power Amplifier Design Using Transistor-Stacking Technique

**Figure 1:**
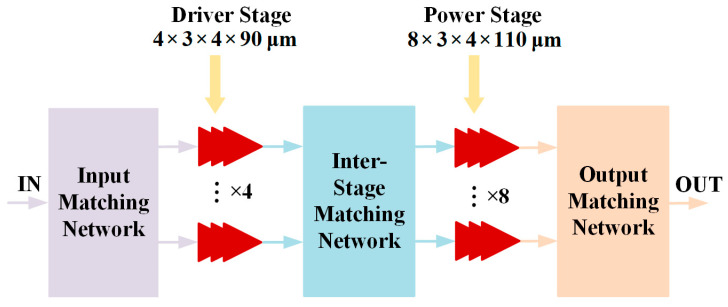
Functional block diagram of the presented SRMPA.

The motivation of this work is to design an ultra-wideband PA that achieves an average output power greater than 40 dBm, a small-signal gain greater than 25 dB, and a high PAE larger than 25%. To achieve this, a power amplifier architecture based on a series stack of multiple GaN-based transistors is proposed, as shown in the topology in Figure 1. In this design, the power stage employs eight-channel 3 × 4 × 110 μm GaN transistors to meet the required high output power, while the driver stage uses four-channel 3 × 4 × 90 μm GaN transistors to ensure sufficient driving power. The input matching and inter-stage matching networks are designed to obtain a good input match and wideband performance, whilst the output matching network is selected to reach a high efficiency and minimized impact on output power.

### 2.1. Transistor-Stacking Technology

Transistor stacking is a technique employed to enhance output power by increasing the voltage swing. However, due to the maximum breakdown voltage of transistors, the voltage swing and the output power are inherently limited. To overcome this limitation, the output power can be increased by stacking transistors in series, thereby expanding the achievable voltage swing.

**Figure 2:**
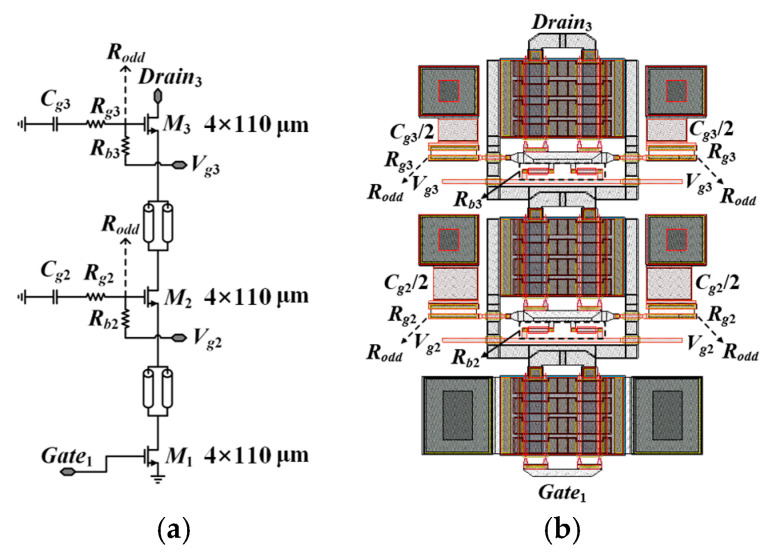
Schematic and layout of the 3 × 4 × 110 μm 3-stacked-FET cell. (**a**) Schematic. (**b**) Layout.

The design of the stacked amplifier unit is the central aspect of this circuit design, as it significantly influences both the output power and gain performance of the amplifier. In this work, a stacked configuration comprising three 4 × 110 μm transistors is utilized. To ensure that each transistor operates at its optimal power performance, the gate bias of each transistor is carefully adjusted to maintain proper operating conditions for all transistors. The circuit diagram of the designed stacked amplifier unit is shown in Figure 2.

To ensure stability and optimize large-signal RF drain voltage distribution, the design employs symmetrically placed gate capacitors (*C_g_*_2_, *C_g_*_3_) and resistors (*R_g_*_2_, *R_g_*_3_) on both sides of the stacked FETs. To suppress odd-mode oscillations over the operating frequency range and improve circuit stability, a resistor *R_odd_* is employed. Under the assumption that parasitic capacitances in the stacked FETs are negligible, the voltage swings of each stacked FET add constructively in phase. Consequently, the input impedance and optimal load impedance of each FET are purely resistive. For the *k*-th stacked FET, the optimal load resistance is *k* times the optimal load resistance of a single FET, expressed as *R_opt,k_* = *k* × *R_opt,_*_1_. Moreover, the input impedance of the *k*-th FET, which corresponds to the load impedance of the (*k* − 1)-th transistor, can be expressed as follows [19]:

(1) $$Z_{in,k}≅\frac{1}{g_{m}}(1+\frac{C_{gm}}{C_{k}}),\;where\;C_{k}=\frac{C_{gs,k}}{g_{m}(k-1)R_{opt,1}-1}$$

**Figure 3:**
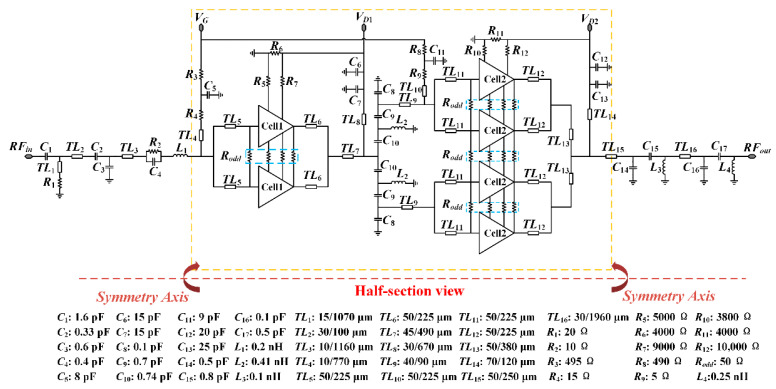
Complete schematic of the presented SRMPA.

Assuming a unilateral transistor configuration, the input impedance is independent of the load impedance. By integrating the simplified equations, the external capacitance at the gate terminal of the CG-FET can be determined, and it is observed that this capacitance decreases as the number of stacked FETs increases. However, in contrast to low-frequency conditions, the optimal load impedance of stacked FETs becomes more complex at high frequencies. Due to the impact of the drain-to-source capacitance, inductive termination (negative reactance) is required. Furthermore, parasitic effects cannot be neglected, which further complicates the high-frequency load impedance characteristics. Specifically, the input admittance *Y_in_*_,*k*_ of the *k*-th stacked FET is determined by the load admittance *Y_L_*_,*k*_ at its output terminal, as detailed in [19].

(2) $$Y_{in,k}=G_{in,k}-jB_{in,k}=\frac{j\omega C_{gs}+g_{m}}{A_{k}}+(\frac{Y_{L,k}-\frac{g_{m}}{A_{k}}}{Y_{L,k}+j\omega C_{ds}})\cdot j\omega C_{ds}$$

where *C_ds_*, *C_gs_*, and *g_m_* are the drain–source capacitance, the gate–source capacitance, and the transconductance of the *k*th transistor, respectively. Due to the presence of these parasitic capacitances, the conjugate complex output impedance is significantly higher than the optimal load impedance in the case of three stacked FETs. Therefore, a trade-off must be made between output power and return loss. The complete schematic of the presented SRMPA is shown in Figure 3. Herein, resistor *R*_5_ is used to connect *V_g_*_2_, and *R*_7_ connects *V_g_*_3_, as shown in Figure 2a. Together, *R*_5_–*R*_7_ provide the appropriate voltages to the cells for proper operation. Similarly, *R*_10_–*R*_12_ perform the same functions at the corresponding stages in the output. The design utilizes a stacked-FET architecture with a symmetrical layout to mitigate parasitic effects and enhance bandwidth. Integrated matching networks and passive components enable high gain and output power over a wide frequency range, maintaining a compact chip size.

### 2.2. Output Matching Network

In the design of the output matching network for 6–18 GHz ultra-wideband applications, four key considerations must be addressed.

(1) The output matching network must convert the 50 ohm impedance to the optimal load impedance required at the output port of the stacked transistors across the 6–18 GHz frequency range, while minimizing the introduced insertion loss (IL).
(2) Broadband characteristic balancing must be considered in the design process. Given that power and efficiency are typically lowest in the frequency band around 18 GHz, a trade-off is necessary by sacrificing low-frequency performance to ensure insertion loss and impedance matching at 18 GHz.
(3) Maintaining appropriate broadband gain flatness is critical to avoid oscillations or phase inconsistencies.
(4) Considering current handling capacity, integrating the bias and bypass networks on-chip is a prudent design choice.

Typically, RF ground microstrip lines or spiral inductors are used to form on-chip RF chokes for DC power supply. In particular, for reactive matching power amplifiers (RMPAs), ground microstrip lines are commonly employed, as they not only facilitate DC feeding but also achieve impedance matching. However, traditional microstrip lines are unsuitable for 10 W 6–18 GHz RMPA designs. This is because the width of the microstrip line must be increased to accommodate higher current handling, which simultaneously increases the parasitic capacitance. Moreover, the length of the microstrip line must be sufficiently long to meet matching requirements at the lowest operation frequency, i.e., 6 GHz. Consequently, as the width and length of the microstrip line increase, the induced parasitic resonant frequency decreases, leading to unwanted resonances within the 6–18 GHz frequency range.

To address this issue, a multi-stage LC structure is conventionally employed after the RF choke in the RMPA to gradually match the load impedance to 50 Ω. The LC structure can improve gain flatness, but increasing the length of the LC matching branches will lead to higher insertion loss. This multi-stage LC approach allows gradual impedance transformation, preventing large parasitic effects from degrading performance.

**Figure 4:**
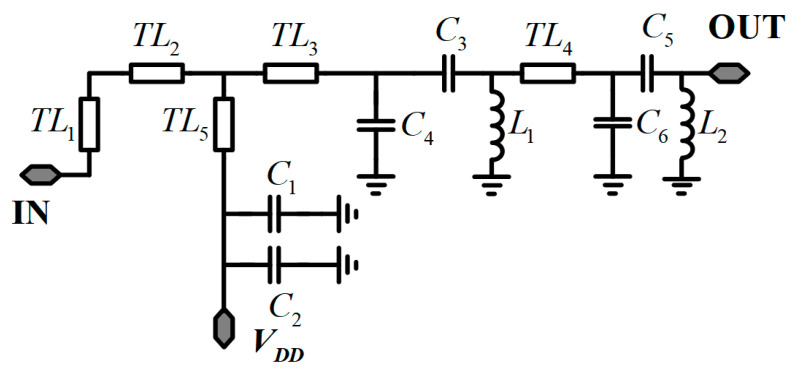
Half layout of the output-matching network.

**Figure 5:**
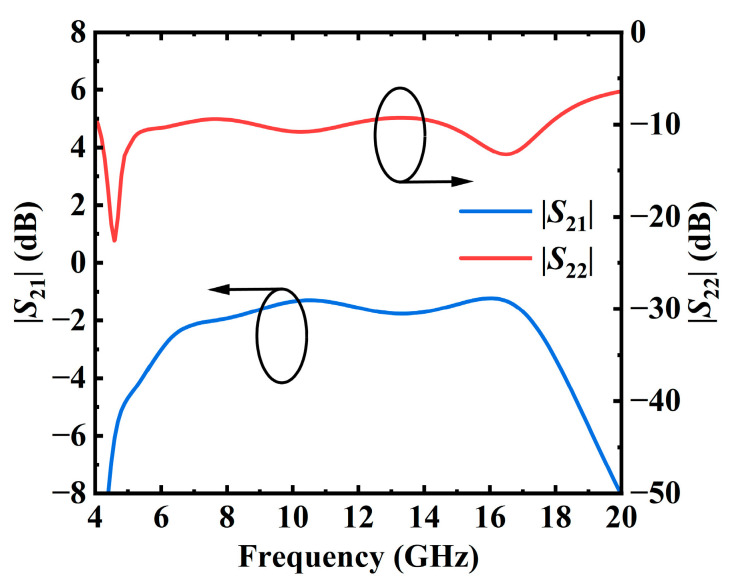
Output return loss and insertion loss of the output-matching network.

To enhance efficiency and minimize impact on output power, an LC reactance structure is utilized, as depicted in Figure 4. Return loss and insertion loss are further optimized through S-parameter and harmonic balance (HB) simulations. Figure 5 presents the output return loss and insertion loss of the output network. These simulations optimize the matching network for low insertion loss across the entire frequency range while ensuring high efficiency. It is seen that the insertion loss of the output matching network is −3.01–−1.22 dB with a return loss better than 10 dB across the frequency range of 6–18 GHz, ensuring high efficiency and high output power of the amplifier.

### 2.3. Interstage Matching Network and Input Matching Network

The interstage matching network is designed to ensure proper impedance matching between the driver and power stages across the 6–18 GHz frequency range, facilitating efficient power transfer and maintaining system stability. Figure 3 illustrates that a combination of grounded inductors and LC matching networks is employed for interstage matching. This approach effectively provides broadband impedance matching and ensures the stability of the interface between the driver and power stages, preventing potential instability arising from impedance mismatches.

In the input matching network design, an RC network is incorporated to improve broadband input matching performance. This configuration effectively reduces the inherent gain of the transistor at lower frequencies, where oscillations are more likely to occur. By integrating the RC network, the input matching network not only enhances broadband matching but also mitigates the risk of low-frequency oscillations, ensuring stable input performance across the operational frequency range. Additionally, an RCL network, consisting of resistors *R*_3_ and *R*_4_, bypass capacitors, and a microstrip line *TL*_4_, is placed within the DC bias path. This network plays a crucial role in preventing thermal runaway by limiting the gate current, ensuring that the transistor operates within safe thermal limits.

## 3. Experimental Results

**Figure 6:**
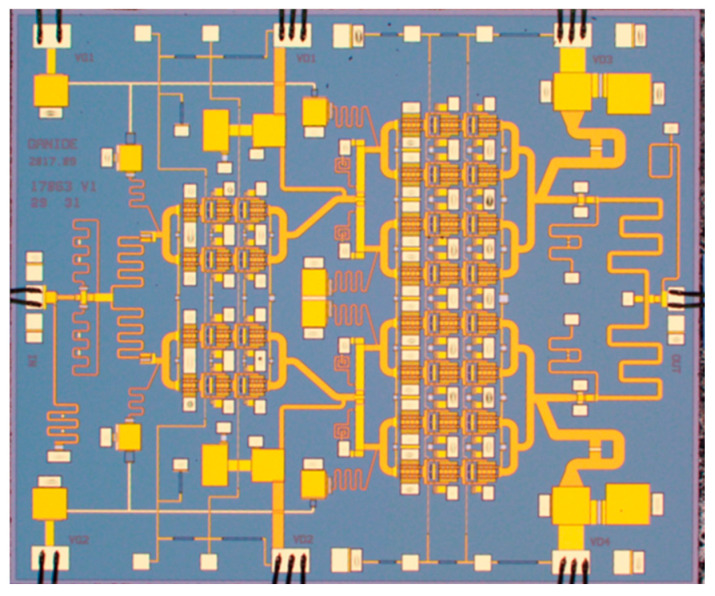
Microphotograph of the GaN SRMPA (4.5 × 3.4 mm^2^).

To verify the effectiveness of the proposed design, a prototype was fabricated using a 0.1 μm GaN-on-Si HEMT process. Figure 6 shows a microphotograph of the MMIC PA. It occupies a footprint size of 4.5 × 3.4 mm^2^ with a Si substrate thickness of 100 μm. The amplifier was measured under CW conditions at an ambient temperature of 25 °C. The gate voltage bias and drain voltage bias are −1.5 V and 28 V, respectively.

**Figure 7:**
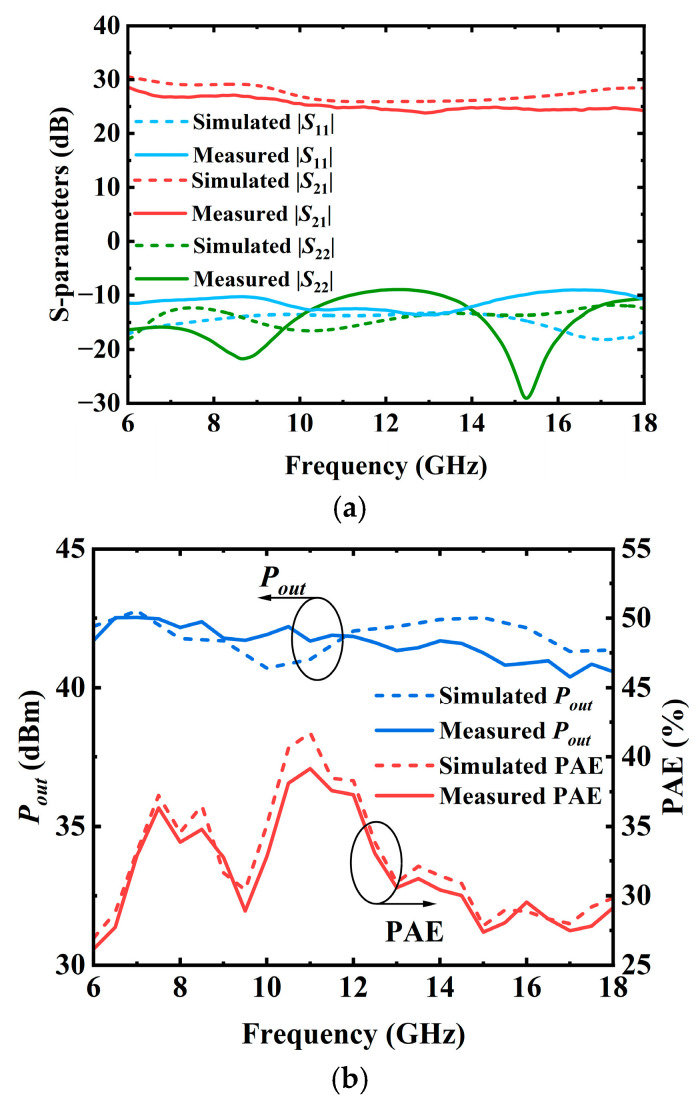
Simulated results of the presented PA. (**a**) S-parameters of the PA. (**b**) *P_out_* and PAE of the PA.

**Figure 8:**
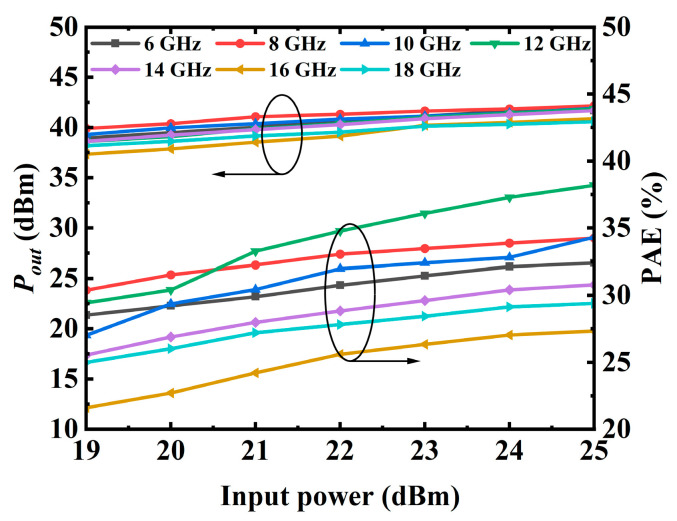
Measured *P_out_* and PAE with input power at 6, 8, 10, 12, 14, 16, and 18 GHz.

Figure 7a shows the simulated and measured S-parameters. It shows that the fabricated PA has a small-signal gain of 25–29 dB, accompanied by an input return loss better than 10 dB and an output return loss larger than 9.5 dB over 6–18 GHz. Large-signal measurements are conducted using CW signals at various frequencies. Figure 7b shows a comparison of the simulated and measured saturated output power and PAE. The PA could achieve an output power (denoted with *P_out_*) of 40.8–42.5 dBm and a PAE of 27–38%, highlighting the PA’s capability to deliver both high output power and high efficiency over a broad frequency range. Figure 8 shows the measured *P_out_* and PAE with input power at a number of frequencies. The results exhibit smooth and monotonic characteristics, with no observable signs of odd-mode or parametric oscillations, indicating robust stability under large-signal operation. In order to directly display the advantages of the presented SRMPA, Table 1 provides a performance comparison with previously reported MMIC PAs. Compared with other counterpart works, the presented SRMPA exhibits competitive performance in terms of gain, output power, and especially PAE, thus demonstrating the rationality of the proposed design approach.

## 4. Conclusions

This article presents the analysis, design, and implementation of a GaN PA operating over the 6–18 GHz frequency range. To enhance output power and efficiency performance, transistor stacking is employed along with a reactive matching network. The PA is fabricated using a 0.1 μm GaN-on-Si HEMT process. The measurement results demonstrate that the chip achieves a small-signal gain of 25–29 dB, a saturated output power of 40.8–42.5 dBm, and a power-added efficiency of 27–38% across the entire band. Experimental results verify the ability of the amplifier to maintain high output power and efficiency over a broad frequency range. The presented chip represents a promising candidate for 6–18 GHz applications.

## Figures and Tables

**Table 1:** Performance comparison with previously reported MMIC PAs.

Ref.	Freq (GHz)	Process	Topology	S21 (dB)	*P_out_* (dBm)	PAE (%)	Size (mm^2^)
[1]	6–18	0.25 μm GaN	NDPA	17–21	41.6–44.2	13.2–23.7	4.8 × 2.3
[4]	2–18	0.25 μm GaN	NDPA	20.8–26	39–41.8	20–28	4.2 × 3.2
[5]	2–18	0.2 μm GaN	DPA	12–13.8	40.2–41.4	20–30	5.0 × 2.5
[8]	6–18	0.15 μm GaN	Class AB	24–34	41.1–44.1	19–40	4.0 × 3.0
[9]	2–18	0.25 μm GaN	DPA	15–23	39.8–41.5	21.8–32	3.6 × 5.0
This work	6–18	0.1 μm GaN	SRMPA	25–29	40.8–42.5	27–38	4.5 × 3.4

Notes: NDPA: nonuniform distributed power amplifier; DPA: distributed power amplifier; SRMPA: stacked reactive matching power amplifier.

## Data Availability

The presented data in this paper are available on request from the corresponding author.
